# A diagnostic scoring model of ENKTCL in the nose-Waldeyer’s ring based on logistic regression: Differential diagnosis from DLBCL

**DOI:** 10.3389/fonc.2023.1065440

**Published:** 2023-02-15

**Authors:** Jun-Yi Xiang, Xiao-Shan Huang, Na Feng, Xiao-Zhong Zheng, Qin-Pan Rao, Li-Ming Xue, Lin-Ying Ma, Ying Chen, Jian-Xia Xu

**Affiliations:** ^1^ Department of Radiology, The Second Affiliated Hospital of Zhejiang Chinese Medical University, Hangzhou, Zhejiang, China; ^2^ Department of Radiology, Second Affiliated Hospital, School of Medicine, Zhejiang University, Hangzhou, Zhejiang, China

**Keywords:** nose-Waldeyer’s ring, computed tomography, MRI, logistic regression, diffuse large B cell lymphoma (DLBCL), extranodal NK/T cell lymphoma (ENKTCL)

## Abstract

**Objective:**

To establish a logistic regression model based on CT and MRI imaging features and Epstein-Barr (EB) virus nucleic acid to develop a diagnostic score model to differentiate extranodal NK/T nasal type (ENKTCL) from diffuse large B cell lymphoma (DLBCL).

**Methods:**

This study population was obtained from two independent hospitals. A total of 89 patients with ENKTCL (n = 36) or DLBCL (n = 53) from January 2013 to May 2021 were analyzed retrospectively as the training cohort, and 61 patients (ENKTCL=27; DLBCL=34) from Jun 2021 to Dec 2022 were enrolled as the validation cohort. All patients underwent CT/MR enhanced examination and EB virus nucleic acid test within 2 weeks before surgery. Clinical features, imaging features and EB virus nucleic acid results were analyzed. Univariate analyses and multivariate logistic regression analyses were performed to identify independent predictors of ENKTCL and establish a predictive model. Independent predictors were weighted with scores based on regression coefficients. A receiver operating characteristic (ROC) curve was created to determine the diagnostic ability of the predictive model and score model.

**Results:**

We searched for significant clinical characteristics, imaging characteristics and EB virus nucleic acid and constructed the scoring system *via* multivariate logistic regression and converted regression coefficients to weighted scores. The independent predictors for ENKTCL diagnosis in multivariate logistic regression analysis, including site of disease (nose), edge of lesion (blurred), T2WI (high signal), gyrus like changes, EB virus nucleic acid (positive), and the weighted score of regression coefficient was 2, 3, 4, 3, 4 points. The ROC curves, AUCs and calibration tests were carried out to evaluate the scoring models in both the training cohort and the validation cohort. The AUC of the scoring model in the training cohort were 0.925 (95% CI, 0.906-0.990) and the cutoff point was 5 points. In the validation cohort, the AUC was 0.959 (95% CI, 0.915-1.000) and the cutoff value was 6 points. Four score ranges were as follows: 0-6 points for very low probability of ENKTCL, 7-9 points for low probability; 10-11 points for middle probability; 12-16 points for very high probability.

**Conclusion:**

The diagnostic score model of ENKTCL based on Logistic regression model which combined with imaging features and EB virus nucleic acid. The scoring system was convenient, practical and could significantly improve the diagnostic accuracy of ENKTCL and the differential diagnosis of ENKTCL from DLBCL.

## 1 Introduction

Extranodal NK/T-cell lymphoma, nasal type (ENKTCL) is a rare subtype of non-Hodgkin lymphoma (NHL) that mostly occurs in the nose-Waldeyer’s ring, accounting for 15% of NHL (1, 2). ENKTCL is highly invasive and prevalent in East Asia and Central and South America. It is closely related to EB virus infection, and its main clinical features are frequent angiocentric necrosis (3). Besides, it has a tendency of extranodal transmission, with rapid progression and poor prognosis. Another major pathological type of NHL at this site is diffuse large B cell lymphoma (DLBCL), which accounts for about 56% of NHL and has a relatively good prognosis (4). It has been demonstrated by several retrospective studies that ENKTCL was more invasive and had poorer prognosis than DLBCL with regards to their clinical features. The incidence, treatment options and prognosis differ between ENKTCL and DLBCL in the nose-Waldeyer’s ring (5, 6). Currently, the standard treatment for ENKTCL is to use a combination of anthracycline-free chemotherapy and radiotherapy, or chemotherapy alone (7, 8), while the most common treatment regimen for DLBCL is R-CHOP (rituximab cyclophosphamide, doxorubicin, vincristine, and prednisone) (8). Therefore, to perform accurate preoperative diagnosis of ENKTCL, and to conduct differential diagnosis of ENKTCL with DLBCL are critically important for identifying the best treatment strategies for ENKTCL.

However, endoscopic excisional biopsy, as the gold standard for definite diagnosis of various diseases, has extremely low sensitivity for the diagnosis of lymphoma in the nose-Waldeyer’s ring (9). Lymphoma originates in the subepithelium, and its lesions may be covered by overlying inflammation, pleomorphic infiltration or extensive necrosis. Particularly, ENKTCL is pathologically characterized by diffuse lymphocytic infiltration, and it progresses with angiocentric and vascular destructive growth, leading to tissue necrosis due to ischemia, and mucosal ulceration and extensive coagulative necrosis. Therefore, surface sampling may lead to the misdiagnosis of deep underlying malignant lymphoma as inflammation (9).

Computed tomography (CT) and magnetic resonance imaging (MRI) have been widely used as the primary imaging modalities for the early diagnosis, staging and treatment evaluation of lymphoma in the paranasal sinuses-Waldeyer’s ring due to their advantages of practicability and convenience (10). In addition, it has been confirmed that MRI is extremely vital to the differential diagnosis of paranasal sinus neoplasms (11, 12), especially to the differential diagnosis of ENKTCL and DLBCL. Most of the previous studies on the diagnosis and differential diagnosis of ENKTCL and DLBCL based on MRI signs were descriptive analysis, and the results could not be obtained directly and conveniently (13). Few studies were reported by establishing a score model for the diagnosis of the disease, which is a method that is simpler, more reliable and can improve the diagnostic accuracy by assigning values based on the imaging features and EB virus of significant diagnostic values following the principle that the higher the diagnostic value, the higher the score.

The objective of this study was to construct a Diagnostic Scoring Model of ENKTCL using logistic regression based on CT and MRI imaging features and EB virus nucleic acid, aiming at improving the diagnostic accuracy of ENKTCL, and the value of differential diagnosis between ENKTCL and DLBCL.

## 2 Materials and methods

### 2.1 Patients

**Figure 1:**
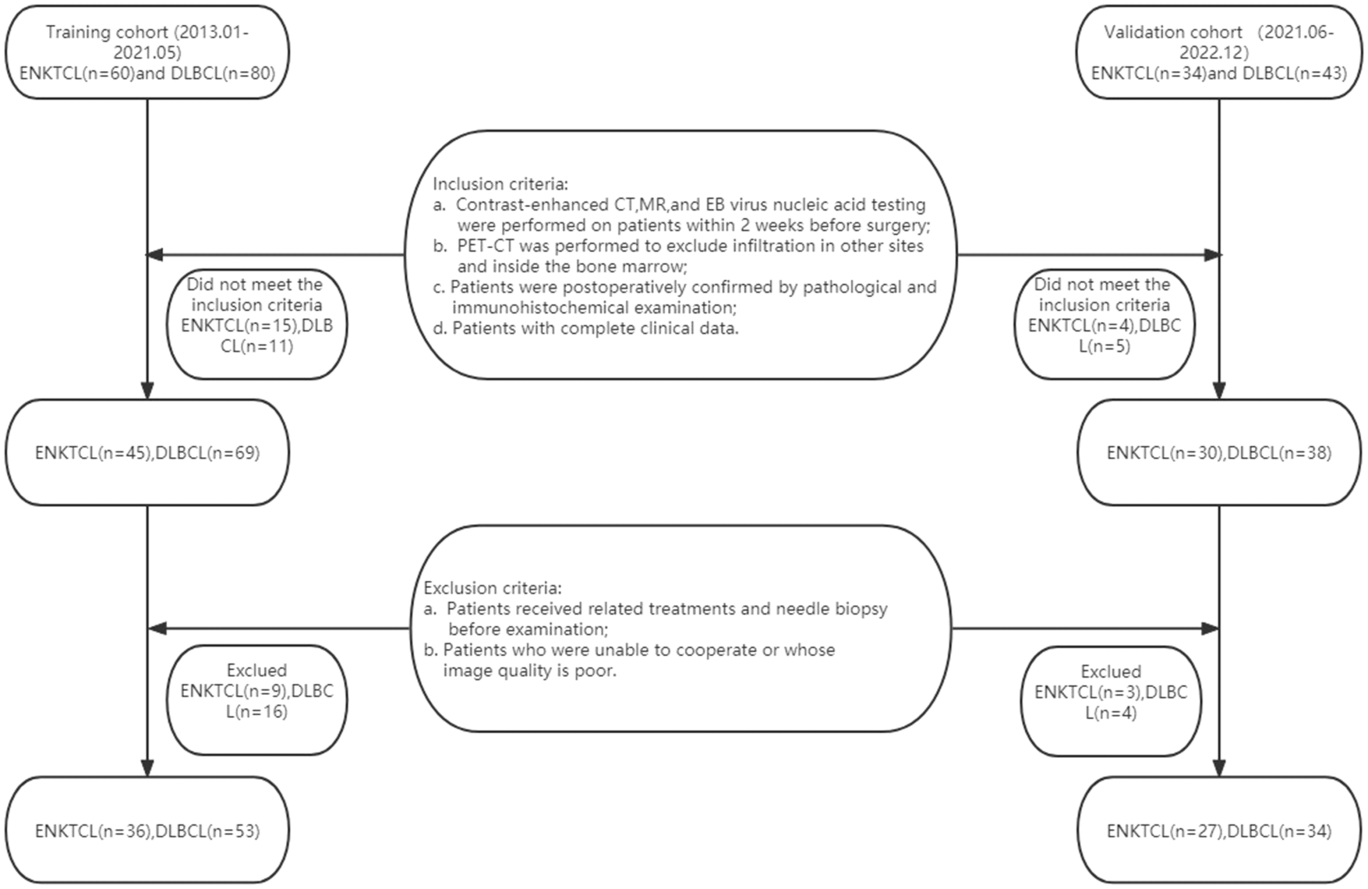
The flowchart of the patient selection.

The institutional review board of our hospital approved this retrospective study and waived informed consent for all patients. This study population was obtained from two independent hospitals. A total of 89 patients with ENKTCL (n = 36) or DLBCL (n = 53) from January 2013 to May 2021 were retrospectively collected as the training cohort in The Second Affiliated Hospital of Zhejiang Chinese Medical University and The Second Affiliated Hospital of Zhejiang University School of Medicine, and 61 patients (ENKTCL=27; DLBCL=34) from Jun 2021 to Dec 2022 were enrolled as the validation cohort. Inclusion criteria: (a) Contrast-enhanced CT, contrast-enhanced MR, and EB virus nucleic acid testing were performed on patients within 2 weeks before surgery; (b) Patients were subjected to Positron Emission Tomography-Computed Tomography (PET-CT) to exclude infiltration in other sites and inside the bone marrow; (c) Patients were postoperatively confirmed by pathological and immunohistochemical examination; (d) Patients with complete clinical data. Exclusion criteria: (a) Patients received related treatments (chemotherapy, radiotherapy, or chemoradiotherapy) and needle biopsy before examination; (b) Patients who were unable to cooperate or whose image quality is poor; (c) Patients in the absence of enhanced CT, enhanced MR, or EB virus nucleic acid testing. 24 patients with ENKTCL and 27 with DLBCL were excluded according to the above criteria, actually leaving 36 patients with ENKTCL and 53 with DLBCL finally included **(** **Figure 1** **)**.

### 2.2 Clinical data and EB virus nucleic acid collection

All patients were performed with required examination. The clinical data and biomarker included age, gender, clinical symptoms (nasal congestion, pharyngeal discomfort, facial discomfort, neck discomfort, eye discomfort), and EB virus nucleic acid (negative, positive) of patients were recorded. EB virus nucleic acid detection whole blood samples were collected and quantitative real-time PCR method was used to detect EBV DNA content in the samples to determine the positive or negative EBV.

### 2.3 Image acquisition

CT examinations were carried out using GE Light speed 16-slice and Siemens SOMATOM Definition 40-slice spiral CT scanners. Axial and coronal plain scanning and enhanced axial venous scanning were performed. Scanning parameters: tube current: 300mA; tube voltage: 120kV; matrix: 512x512; layer thickness: 3mm; window width: 250HU; and window level: 50HU. MR examinations were conducted using Siemens 1.5T, GE 3.0T magnetic resonance scanners, and quadrature head coil. Axial and coronal plain scanning, and enhanced axial, coronal and sagittal scanning were performed. Scanning parameters: T1WI: TR=230, TE=2.30; T2WI: TR=2800, TE=82.12; STIR sequences in coronal positions: TR=3200, TE=2.70; matrix: 256x256, layer thickness: 4mm, and interval: 1mm. Contrast-enhanced CT and MR scanning were respectively administered with the contrast agents of non-ionic iodine (ioversol, iohexol, dosage of 1.5ml/kg, flow rate of 3ml/s) and Gd-DTPA (dosage of 0.1mmol/kg, flow rate of 2ml/s) intravenously injected through the cubital vein with high-pressure syringe. The scanning range of CT and MR: the axial scanning was from the upper edge of the frontal sinus to the lower edge of the second cervical vertebra, while the coronal scanning was from the frontal sinus to the posterior edge of the sphenoid sinus, and the sagittal scanning covered the whole nasal cavity and paranasal sinuses.

### 2.4 Image analysis

The CT and MR images of all patients were retrospectively reviewed and analyzed by two doctors (having 11- and 20-year diagnostic experience, respectively) from the Department of Head & Neck Radiology, and consensus was reached through discussion when disagreements arose. All assessments were performed blindly. The main contents of image analysis are listed as follows: (1) Site (waldeyer’s ring, nose); (2) Distribution (right, left or bilateral sides); (3) Range (diffuse, limit); (4) Edge (clear, blurred); (5) Density/signal (heterogeneous, homogeneous); (6) Hemorrhage (yes/no); (7) Cystic (yes/no, non-enhanced low-density lesions with CT value < 20); (8) Pharyngeal recess involvement (yes/no); (9) Carotid artery involvement (yes/no); (10) Sinus complex involvement (yes/no); (11) Bone destruction (yes/no); (12) Tonsil enlargement (yes/no); (13) Cervical lymph node enlargement (yes/no; the shortest diameter≥10cm); (14) Maximum diameter (the maximum cross-sectional diameter at the axial position); (15) Non-enhancement CT (measurement was performed at the central position of the largest section of the tumor, avoiding the sites of necrosis and cystic, hemorrhage, and calcification). (16) Enhancement CT (measurement was performed at the central position of the largest section of the tumor, avoiding the sites of necrosis and cystic, hemorrhage, and calcification in arterial phase). (17) D-value (the arterial phase values minus the non-enhanced phase values); (18) T1WI (iso-intense signal, slightly lower signal, referring to the signal of adjacent muscles); (19) T2WI (slightly high-intense signal and high-intense signal, referring to the signals of adjacent muscles); (20) Gyrus like changes (yes/no); (21) Degree of MR enhancement (mild, moderate, obvious).

All imaging features from the two radiologists were used to assess the interobserver agreement. To assess the intraobserver agreement, the first radiologist re-assessed the abovementioned indexes 2 weeks later.

### 2.5 Statistical analysis

The intra- and interobserver agreements in imaging features were assessed with interclass correlation coefficient (ICC) in the training cohort. ICC values between 0.61 and 0.80, and greater than 0.80 are indicative of good and excellent agreements, respectively; otherwise, the agreement is unsatisfactory (14).

According to the nature and number of covariates, different methods were used to correct covariates. When the main outcome variable was continuity index, difference method or covariance analysis can be used. Hierarchical analysis can be used when the main outcome variables and covariables were categorical indicators. Data distributions were measured using the Kolmogorov-Smirnov test or the Shapiro-Wilk test. Continuous data with normal distribution are shown as mean ± standard deviation and data with a non-normal distribution are shown as median (interquartile range). One-way Analysis of Variance (ANOVA) was performed if the variance was homogeneous, while Kruskal-Wallis H test was conducted if the variance was hierarchical. In univariate analysis, independent-sample t test was used to measure and calculate the continuous variables between the two groups. Categorical variables between the two groups were measured using the chi-square test or Fisher’s exact test. P < 0.05 indicated that the difference was statistically significant. Statistically significant variables were analyzed using multivariate logistic regression in a reverse stepwise way to identify independent predictors of ENKTCL. To obtain an optimal score that is easy to calculate, we converted the regression coefficients to weighted scores by dividing the regression coefficients for each independent predictor by 1/2 of the smallest coefficient, rounding it to the nearest integer or taking the integer part. The scores of independent predictors of each patient were added, and a total score was obtained ranging from 0 to 16 points. According to the score distribution, they were divided into 4 groups, and the diagnostic accuracy of ENKTCL in each distribution area were shown. The goodness of fit of the logical model was calibrated and assessed using Hosmer-Lemeshow test. The area under the ROC curve was used to evaluate the diagnostic efficacy of the prediction model and the score model, and the accuracy, sensitivity, specificity and optimal cutoff point were recorded. The maximum sum of sensitivity and specificity was defined as the optimal cutoff point. All statistical analyses were carried out using SPSS software (IBM SPSS Statistics 25.0, IBM).

## 3 Results

### 3.1 Clinical characteristics and image values

In this study, a total of 89 patients, comprising 36 with ENKTCL and 53 with DLBCL were enrolled as the training cohort. The mean age of patients with ENKTCL was (55.42 ± 10.13) years, and that of patients with DLBCL was (60.16 ± 10.48) years. No significant difference was found between the two groups (P=0.620). The male-to-female ratio of patients with ENKTCL was 29:7, and that of patients with DLBCL was 26:27, indicating statistically significant differences between the two groups (P=0.003). The initial clinical symptoms of patients with ENKTCL: 27 cases presented with nasal congestion, 5 with pharyngeal discomfort, 4 with facial discomfort, 6 with neck discomfort, and 5 with eye discomfort. The difference demonstrated was statistically significant (P<0.001). Among the 36 patients with ENKTCL, 26 (72.22%) were tested positive for EB virus nucleic acid, while only 13 (24.53%) of 53 patients with DLBCL were tested positive, and the difference was statistically significant (P< 0.001) (**Table 1**).

**Table 1:** Clinical characteristics of ENKTCL and DLBCL in the nose-Waldeyer’s ring of the training cohort: univariate analysis.

Clinical characteristics	ENKTCL (n=36)	DLBCL (n=53)	P^*^
Age	55.97 ± 10.13	60.16 ± 10.48	0.620
gender			***0.003***
Male	29 (80.56%)	26 (49.06%)	
Female	7 (19.44%)	27 (50.94%)	
Symptoms			***<0.001***
Nasal obstruction	27 (75.00%)	10 (18.87%)	
Pharyngeal discomfort	5 (13.89%)	23 (43.40%)	
Facial discomfort	4 (11.11%)	9 (16.98%)	
Neck discomfort	0 (0.00%)	6 (11.32%)	
Eye discomfort	0 (0.00%)	5 (9.43%)	
EB virus nucleic acid			***<0.001***
Positive	10 (27.78%)	40 (75.47%)	
Negative	26 (72.22%)	13 (24.53%)	

^*^ P values ≤ 0.05 written in bold and italics indicates a statistically significant difference between two groups.

There were 61 patients were studied in the validation cohort, which contained 27 with ENKTCL and 34 with DLBCL. **Table 2** showed there were significant difference in the Sex, Symptoms or EB virus nucleic acid in the validation cohort according to the univariate analysis (P< 0.05).

**Table 2:** Clinical characteristics of ENKTCL and DLBCL in the nose-Waldeyer’s ring of the validation cohort: Univariate analysis.

Clinical characteristics	ENKTCL (n=27)	DLBCL (n=34)	P^*^
Age	60.00 ± 14.07	63.91 ± 12.29	0.620
gender			***0.040***
Male	19 (70.37%)	15 (44.12%)	
Female	8 (29.63%)	19 (55.88%)	
Symptoms			***0.009***
Nasal obstruction	17 (62.96%)	7 (20.59%)	
Pharyngeal discomfort	4 (14.81%)	14 (41.18%)	
Facial discomfort	3 (11.11%)	3 (8.81%)	
Neck discomfort	2 (7.41%)	5 (14.71%)	
Eye discomfort	1 (3.71%)	5 (14.71%)	
EB virus nucleic acid			***<0.001***
Positive	7 (25.93%)	24 (70.59%)	
Negative	20 (74.07%)	10 (29.41%)	

^*^ P values ≤ 0.05 written in bold and italics indicates a statistically significant difference between two groups.

The CT and MR imaging features of patients with ENKTCL and DLBCL in the training cohort were shown in **Table 3**. Differences in lesion distribution and range, density/signal, hemorrhage, cystic, pharyngeal recess involvement, carotid artery involvement, maximum diameter, D-value, and degree of enhancement between the two groups of patients with different pathological types were not statistically significant (P>0.05); while differences in site, edge, sinus complex involvement, bone destruction, tonsil enlargement, cervical lymph node enlargement, non-enhancement CT, enhancement CT, signals on T1WI and T2WI, and gyrus like changes between the two groups were statistically significant (P<0.05). The same comparison and analysis were performed in the validation cohort. All relevant predictors (P<0.05) in the training cohort maintained statistical difference in the validation cohort apart from bone destruction, tonsil enlargement and T1WI (**Table 4**).

**Table 3:** Imaging features comparison among ENKTCL and DLBCL in the nose-Waldeyer’s ring of the training cohort: univariate analysis.

Image characteristics	ENKTCL (n=36)	DLBCL(n=53)	P^*^
Site			***<0.001***
Waldeyer’s ring	10 (27.78%)	35 (66.04%)	
Nose	26 (72.22%)	18 (33.96%)	
Distribution			0.799
Right	14 (38.89%)	17 (32.08%)	
Left	15 (41.67%)	25 (47.17%)	
Bilateral sides	7 (19.44%)	11 (20.75%)	
Range			0.260
Diffuse	20 (55.56%)	23 (43.40%)	
Limit	16 (44.44%)	30 (56.60%)	
Edge			***0.001***
Clear	3 (8.33%)	21 (39.62%)	
Blurred	33 (91.67%)	32 (60.38%)	
Density/Signal			0.537
Heterogeneous	10 (27.78%)	18 (33.96%)	
Homogeneous	26 (72.22%)	35 (66.04%)	
Hemorrhage			0.142
No	27 (97.22%)	41 (88.68%)	
Yes	9 (2.78%)	12 (11.32%)	
Cystic			0.797
No	33 (91.67%)	39 (73.58%)	
Yes	3 (8.33%)	14 (26.42%)	
Pharyngeal recess involvement			0.693
No	23 (63.89%)	36 (67.92%)	
Yes	13 (36.11%)	17 (32.08%)	
Carotid artery involvement			0.768
No	32 (88.89%)	46 (86.79%)	
Yes	4 (11.11%)	7 (13.21%)	
Sinus complex involvement			***0.001***
No	18 (50.00%)	44 (83.02%)	
Yes	18 (50.00%)	9 (16.98%)	
Bone destruction			***0.002***
No	13 (36.11%)	37 (69.81%)	
Yes	23 (63.89%)	16 (30.19%)	
Tonsil enlargement			***0.040***
No	28 (77.78%)	30 (56.60%)	
Yes	8 (22.22%)	23 (43.40%)	
Cervical lymph node involvement			***0.011***
No	28 (77.78%)	27 (50.94%)	
Yes	8 (22.22%)	26 (49.06%)	
Maximum diameter	43.57 ± 17.63	40.18 ± 13.20	0.303
Non-enhancement CT	37.57 ± 6.74	50.13 ± 9.90	***0.001***
Enhancement CT	55.54 ± 8.13	68.53 ± 12.75	***0.001***
D-value	18.00 ± 4.82	18.40 ± 8.82	0.796
T1WI			***<0.001***
Equal signal	6 (16.67%)	39 (73.58%)	
Slightly lower signal	30 (83.33%)	14 (26.42%)	
T2WI			***<0.001***
Slightly higher signal	8 (22.22%)	39 (73.58%)	
High signal	28 (77.78%)	14 (26.42%)	
Gyrus like changes			***0.001***
No	19 (52.78%)	45 (84.91%)	
Yes	17 (47.22%)	8 (15.09%)	
Degree of enhancement			0.054
Low	16 (44.44%)	31 (58.49%)	
Intermediate	18 (50.00%)	14 (26.42%)	
High	2 (5.56%)	8 (15.09%)	

^*^ P values ≤ 0.05 written in bold and italics indicates a statistically significant difference between two groups.

**Table 4:** Imaging features comparison among ENKTCL and DLBCL in the nose-Waldeyer’s ring of the validation cohort: univariate analysis.

Image characteristics	ENKTCL (n=27)	DLBCL (n=34)	P^*^
Site			***0.015***
Waldeyer ring	9 (33.33%)	22 (64.71%)	
Nose	18 (66.67%)	12 (35.29%)	
Distribution			0.197
Right	12 (44.44%)	9 (26.47%)	
Left	7 (25.93%)	16 (47.06%)	
Bilateral sides	8 (29.63%)	9 (26.47%)	
Range			0.126
Diffuse	18 (66.67%)	16 (47.06%)	
Limit	9 (33.33%)	18 (52.94%)	
Edge			***<0.001***
Clear	4 (14.81%)	20 (58.82%)	
Blurred	23 (85.19%)	14 (41.18%)	
Density/Signal			0.251
Heterogeneous	10 (37.04%)	8 (23.53%)	
Homogeneous	17 (62.96%)	26 (76.47%)	
Hemorrhage			0.369
No	27 (100.00%)	33 (97.06%)	
Yes	0 (0.00%)	1 (2.94%)	
Cystic			0.060
No	21 (77.78%)	32 (94.12%)	
Yes	6 (22.22%)	2 (5.88%)	
Pharyngeal recess involvement			0.157
No	19 (70.37%)	29 (85.29%)	
Yes	8 (29.63%)	5 (14.71%)	
Carotid artery involvement			0.055
No	26 (96.30%)	20 (58.82%)	
Yes	1 (3.70%)	14 (41.18%)	
Sinus complex involvement			***<0.001***
No	10 (37.04%)	32 (94.12%)	
Yes	17 (62.96%)	2 (5.88%)	
Bone destruction			0.854
No	17 (62.96%)	23 (67.65%)	
Yes	10 (37.04%)	11 (32.35%)	
Tonsil enlargement			0.147
No	22 (81.48%)	22 (64.71%)	
Yes	5 (18.52%)	12 (35.29%)	
Cervical lymph node involvement			***0.002***
No	21 (77.78%)	23 (67.65%)	
Yes	6 (22.22%)	11 (32.35%)	
Maximum diameter	39.31 ± 15.34	36.69 ± 13.26	0.478
Non-enhancement CT	44.00 ± 5.65	48.96 ± 3.84	***<0.001***
Enhancement CT	63.42 ± 7.48	68.26 ± 4.87	***0.003***
D-value	19.43 ± 6.75	19.18 ± 4.18	0.872
T1WI			0.568
Equal signal	10 (37.04%)	21 (61.76%)	
Slightly lower signal	17 (62.96%)	13 (38.24%)	
T2WI			***<0.001***
Slightly higher signal	6 (22.22%)	23 (67.65%)	
High signal	21 (77.78%)	11 (32.35%)	
Gyrus like changes			***<0.001***
No	12 (44.44%)	30 (88.24%)	
Yes	15 (55.56%)	4 (11.76%)	
Degree of enhancement			***0.045***
Low	2 (7.40%)	3 (8.82%)	
Intermediate	18 (66.67%)	12 (35.30%)	
High	7 (25.93%)	19 (55.88%)	

^*^ P values ≤ 0.05 written in bold and italics indicates a statistically significant difference between two groups.

### 3.2 Establishment of a predictive model

Through univariate analysis (**Tables 1**, **3**) of the training cohort, statistically significant (P<0.05) clinical features and imaging features (gender, clinical symptoms, site, edge, sinus complex involvement, bone destruction, tonsil enlargement, cervical lymph node enlargement, non-enhancement CT, enhancement CT, signals on T1WI and T2WI, and gyrus like changes) as well as positive EB virus nucleic acid were screened out and included in multivariate logistic regression analysis. In the same way, the relevant predictors (P<0.05) with statistical differences in univariate analysis of the validation cohort **(** **Tables 2**, **4** **)** were screened out and included in multivariate logistic regression analysis.

Five independent predictors were identified for the diagnosis of ENKTCL (**Tables 5**, **6**). For training cohort, five independent predictors which included site (odds ratio (OR), 4.384; 95% confidence interval (CI), 0.981-19.590; P=0.045), blurred edge (OR, 8.129; 95% CI, 1.238-53.386; P=0.029), high-intense signal on T2WI (OR, 22.265; 95% CI, 1.238-53.386; P=0.029), gyrus like changes (OR, 9.636; 95% CI, 3.834-51.907; P=0.008) and positive EB virus nucleic acid (OR, 19.953; 95% CI, 3.834-103.841; P=0.008), as presented in **Table 5**; For internal validation cohort, five independent predictors which included site (OR, 3.849; 95% CI, 0.375-39.459; P=.015), blurred edge (OR, 9.801; 95% CI, 0.982-97.854; P=.009), high-intense signal on T2WI (OR, 25.393; 95% CI, 5.493-100.382; P=0.001), gyrus like changes (OR, 9.936; 95% CI, 1.305-41.998; P<0.001) and positive EB virus nucleic acid (OR, 23.515; 95% CI, 3.801-97.854; P <0.001), as presented in **Table 6**; The logistic prediction model for the diagnosis of ENKTCL was constructed accordingly. The results of Hosmer-Lemeshow chi-square test (χ^2 ^= 7.202, P = 0.515; χ^2 ^= 5.306, P = 0.652) indicated that the multivariate logistic model established based on our research data fits well with the real data and is authentic and reliable to reflect the relationship between real variables.

**Table 5:** Multivariate regression analysis for ENKTCL diagnosis in the nose-Waldeyer’s ring and the weighted scores of independent predictors in the training cohort.

	B	P^*^	OR	95% CI		Weighted score
				Lower bound	Upper bound	
Site(Nose)	1.478	***.045***	4.384	.981	19.590	2
Edge ( Blurred )	2.095	***.029***	8.129	1.238	53.386	3
T2WI (High signal)	3.103	**<0.001**	22.265	4.160	119.153	4
Gyrus like changes	2.266	***.008***	9.636	1.789	51.907	3
EB virus nucleic acid	2.993	**<0.001**	19.953	3.834	103.841	4
Constant	-11.273	***.000***	.000			

^*^ P values ≤ 0.05 written in bold and italics indicates a statistically significant difference between two groups.

**Table 6:** Multivariate regression analysis for ENKTCL diagnosis in the nose-Waldeyer’s ring and the weighted scores of independent predictors in the validation cohort.

	B	P^*^	OR	95% CI		Weighted score
				Lower bound	Upper bound	
Site (Nose)	1.348	***.015***	3.849	0.375	39.459	2
Edge ( Blurred )	2.282	***.009***	9.801	0.982	97.854	3
T2WI (High signal)	3.001	**0.001**	25.393	5.493	100.382	4
Gyrus like changes	2.230	**<0.001**	9.936	1.305	41.998	3
EB virus nucleic acid	2.901	**<0.001**	23.515	3.801	97.854	4
Constant	18.574	***.000***	.000			

*P values ≤ 0.05 written in bold and italics indicates a statistically significant difference between two groups.

### 3.3 Establishment of score model

**Figure 2:**
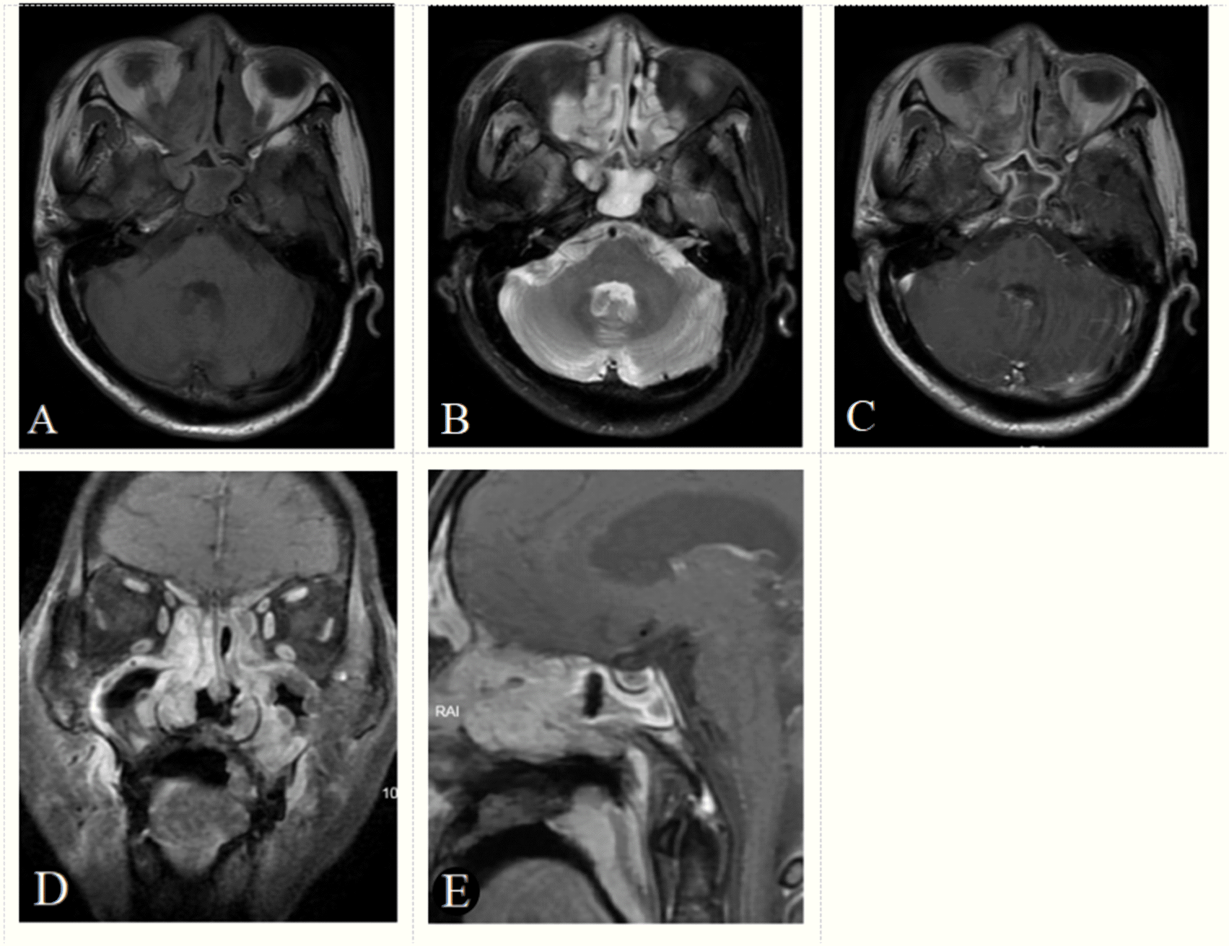
A 70-year-old male patient with positive EB virus nucleic acid and ENKTCL. The tumor appears as a diffuse growth of soft tissue masses in the bilateral nasal cavity and paranasal sinuses, with an isointense signal on axial T1-weighted image **(A)**, a hyperintense signal (signal between muscle and nasal mucosa) on T2-weighted image **(B)**, a heterogeneous enhancement on axial, coronal, sagittal enhancement T1-weighted image **(C–E)** and a gyrus like changes on sagittal enhancement T1-weighted image **(E)**. The tumor boundaries are blurred and the tumor invades the maxilla and adjacent soft tissues. Based on the score model, this patient scored 16 points, the maximum in the points-scoring system.

**Figure 3:**
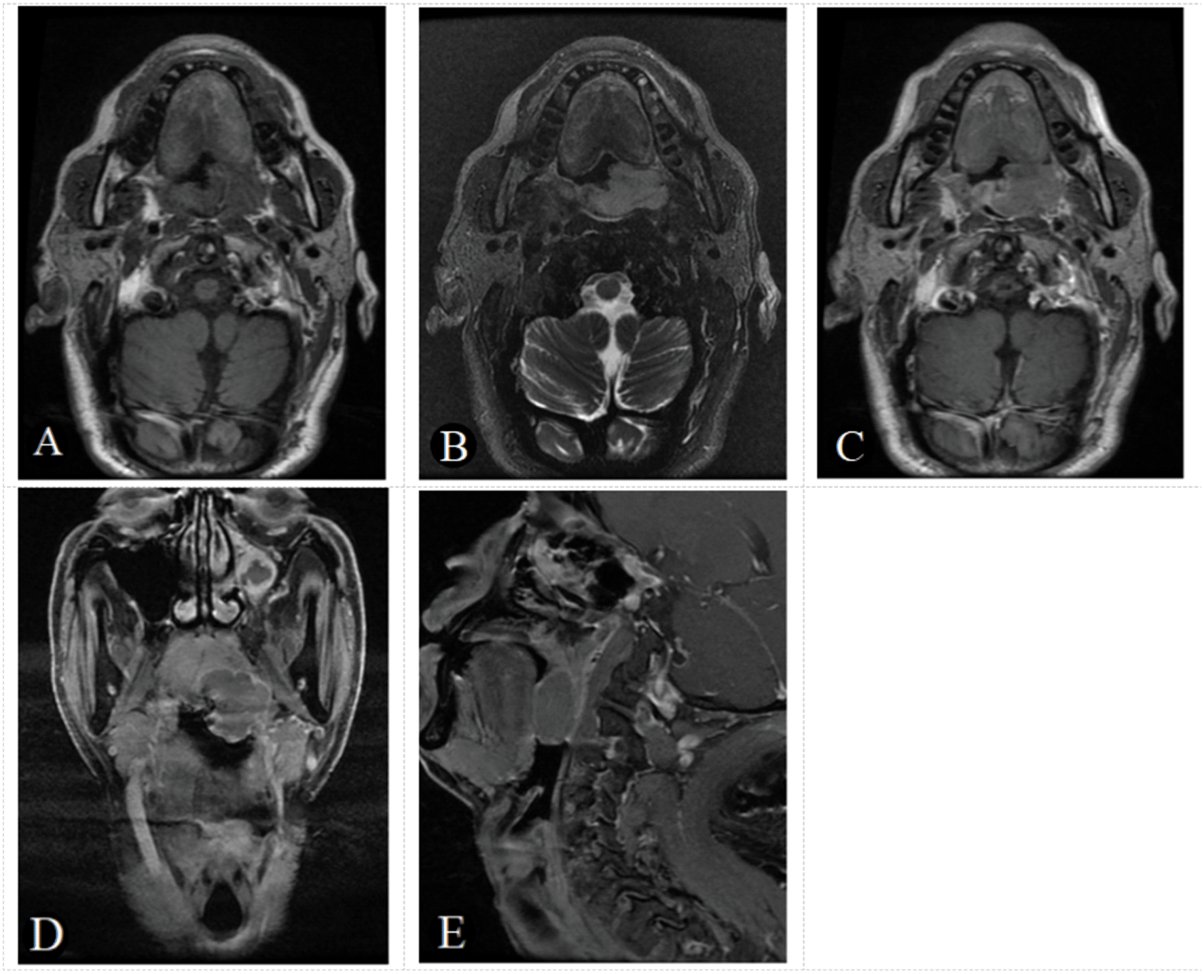
A 61-year-old male patient with negative EB virus nucleic acid and DLBCL in the left oropharynx. The tumor appears as a homogeneous focal nodular with an isointense signal on axial T1-weighted image **(A)**, a slightly hyperintense signal on T2-weighted image **(B)** and a slightly homogeneous enhancement, on axial, coronal, sagittal enhancement T1-weighted image **(C-E)**. The tumor boundaries are clear and there is no bone destruction. Based on the score model, this patients scored 0 points, the minimum in the points-scoring system.

A diagnostic scoring model of ENKTCL was built after the score of each independent predictor was weighted, and the results were described as follows: site (nose): 2 points; blurred edge: 3 points; high-intense signal on T2WI: 4 points; gyrus like changes: 3 points; positive EB virus nucleic acid: 4 points (**Tables 5**, **6**). The scores of independent predictors of each patient were added, and a total score was obtained ranging from 0 to 16 points (**Figures 2**, **3**). The median score in this study was 8, and the extreme scores were 0 and 16 (**Figures 2**, **3**).

### 3.4 Predictive performance of model in the training cohort

**Figure 4:**
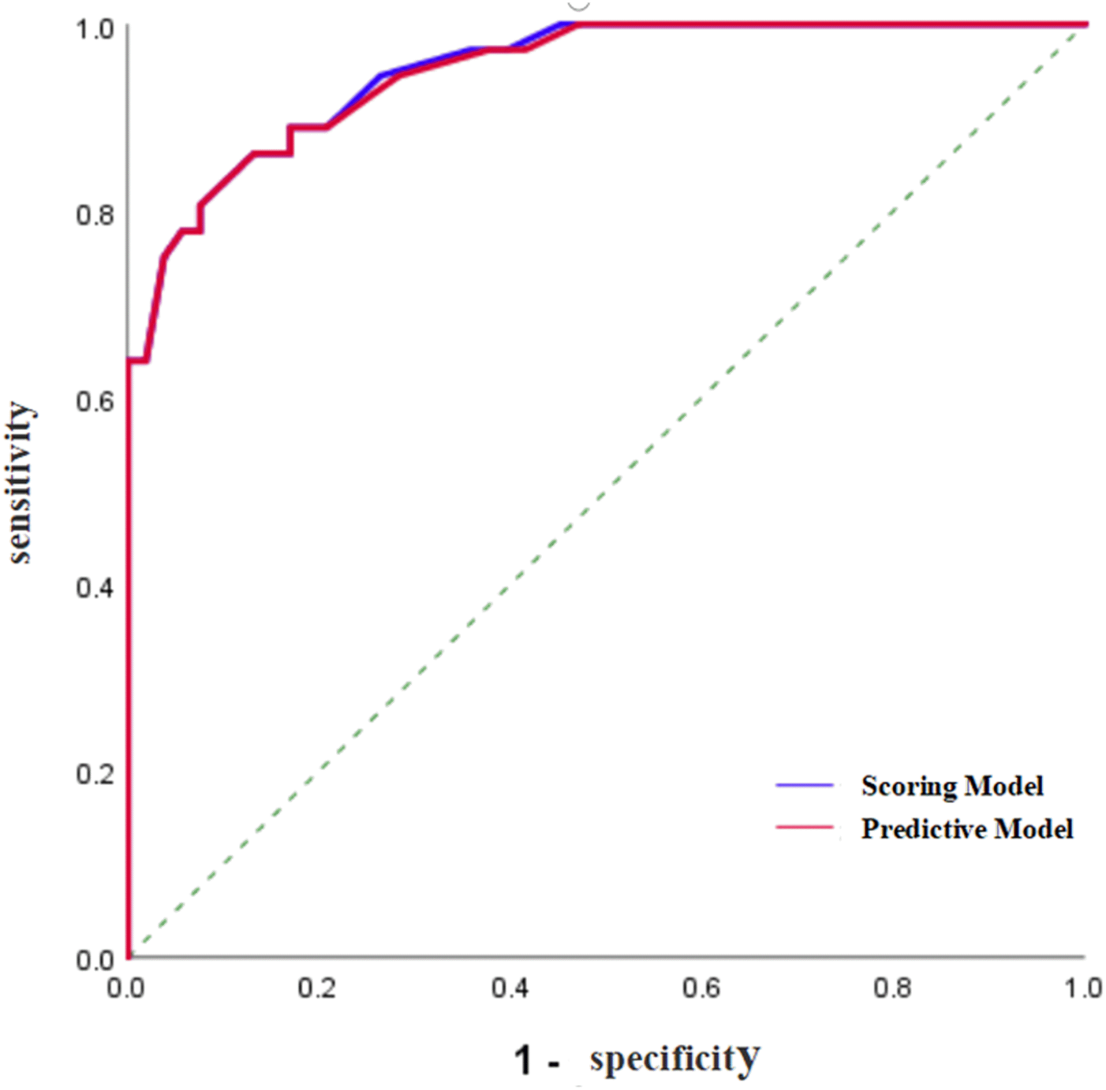
ROC curve of the prediction model and the score model for the diagnosis of ENKTCL in the nose-Waldeyer’s ring of the training cohort.

**Figure 5:**
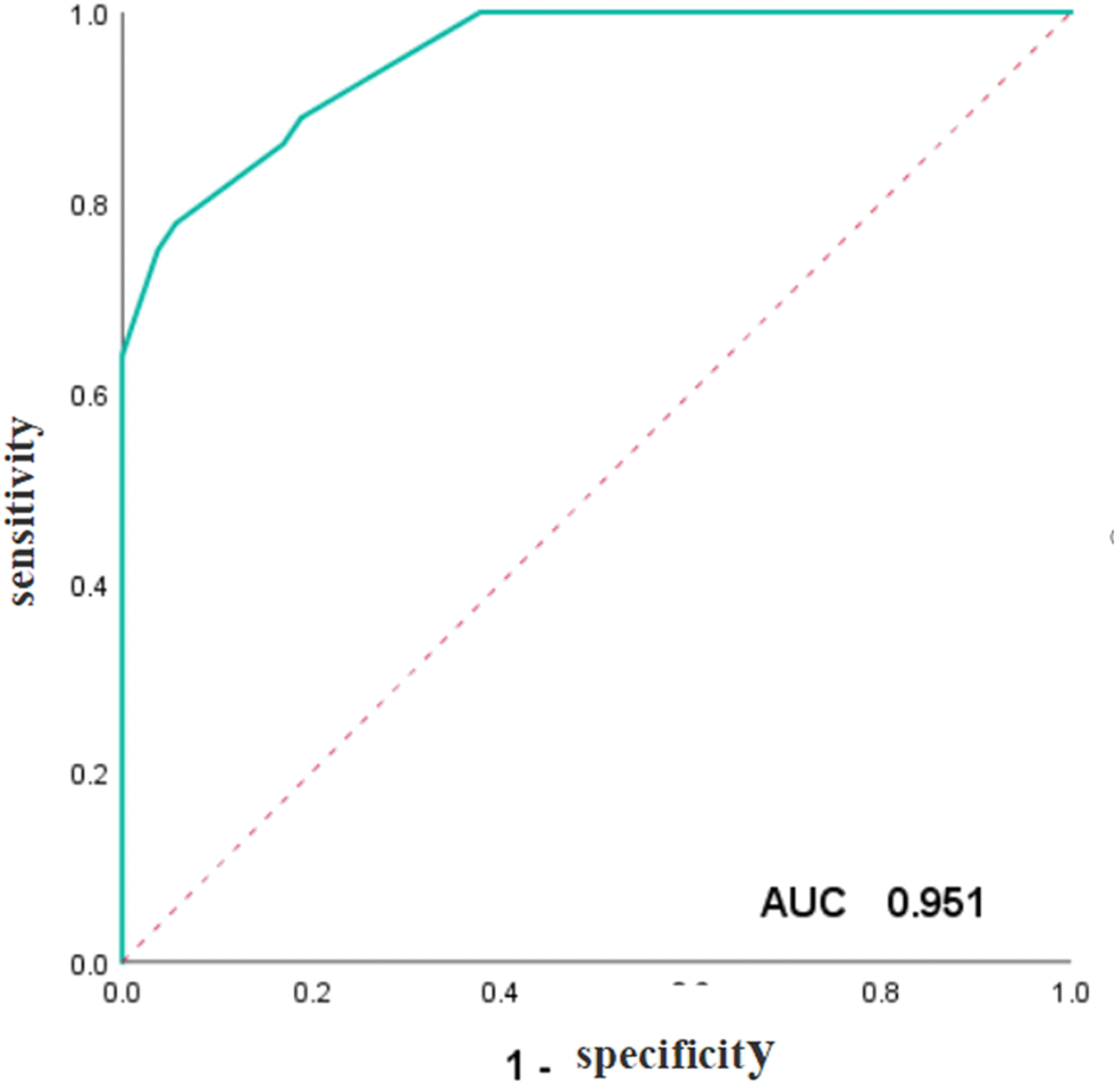
ROC curve of the weighted scores of independent predictive variables of ENKTCL in the nose-Waldeyer’s ring of the training cohort.

To validate and compare the diagnostic efficacy of predictive and scoring model, we performed ROC analysis and calculated their AUCs **(** **Figure 4** **)**. In the training cohort, no statistical difference was demonstrated in the AUCs between the two models (P=0.743), indicating that the score model made full use of the information of the prediction model, and was simple, practical for the diagnosis of ENKTCL by distinguishing it from DLBCL. The AUC of the score model was 0.948 (95% CI, 0.906~0.990; P<0.001), which was close to that of the prediction model whose AUC was 0.949 (95% CI, 0.909~0.990; P<0.001). According to the optimal value of AUC in the training cohort, when the value is assigned to 10, a relatively high diagnostic efficacy can be achieved, with a sensitivity of 77.8%, a specificity of 94.3% and an accuracy of 91.7% (**Table 7**; **Figure 5**).

**Table 7:** Predictive efficacy of the prediction model, the score model and weighted scores for ENKTCL in the nose-Waldeyer’s ring.

Variable	cut-off point	Youden index	Sensitivity	Specificity	AUC	95% CI	P^*^
Predictive model(training)	0.504	0.731	0.806	0.925	0.949	0.909~0.990	***<0.001***
Scoring model(training)	0.508	0.732	0.807	0.925	0.948	0.906~0.990	***<0.001***
Predictive model(validation)	0.533	0.838	0.926	0.912	0.967	0.927-1.000	***<0.001***
Scoring model(validation)	0.614	0.741	0.864	0.877	0.959	0.915-1.000	***<0.001***
Weighted score	10	0.721	0.778	0.943	.951	0.912-0.990	***<0.001***

^*^ P values ≤ 0.05 written in bold and italics indicates a statistically significant difference between two groups.

### 3.5 Score ranges exploration

To facilitate the application of this points-scoring system in radiodiagnosis, we further divided the scores into 4 separate groups: 0-6 points, 7-9 points, 10-11 points, and 12-16 points. Statistically significant difference was shown among the 4 groups in the diagnostic accuracy of ENKTCL (P < 0.001), and an increasing trend of accuracy was revealed with the increase of scoring stages (0%, 33.33%, 82.35% and 100%, respectively) (**Table 8**).

**Table 8:** Four score intervals of ENKTCL in the nose-Waldeyer’s ring.

Score range	ENKTCL(n)	Sum (n)	Diagnostic rate (%)
0-6	0	34	0%
7-9	8	24	33.33%
10-11	14	17	82.35%
12-16	14	14	100%

### 3.6 External validation of the established scoring model

**Figure 6:**
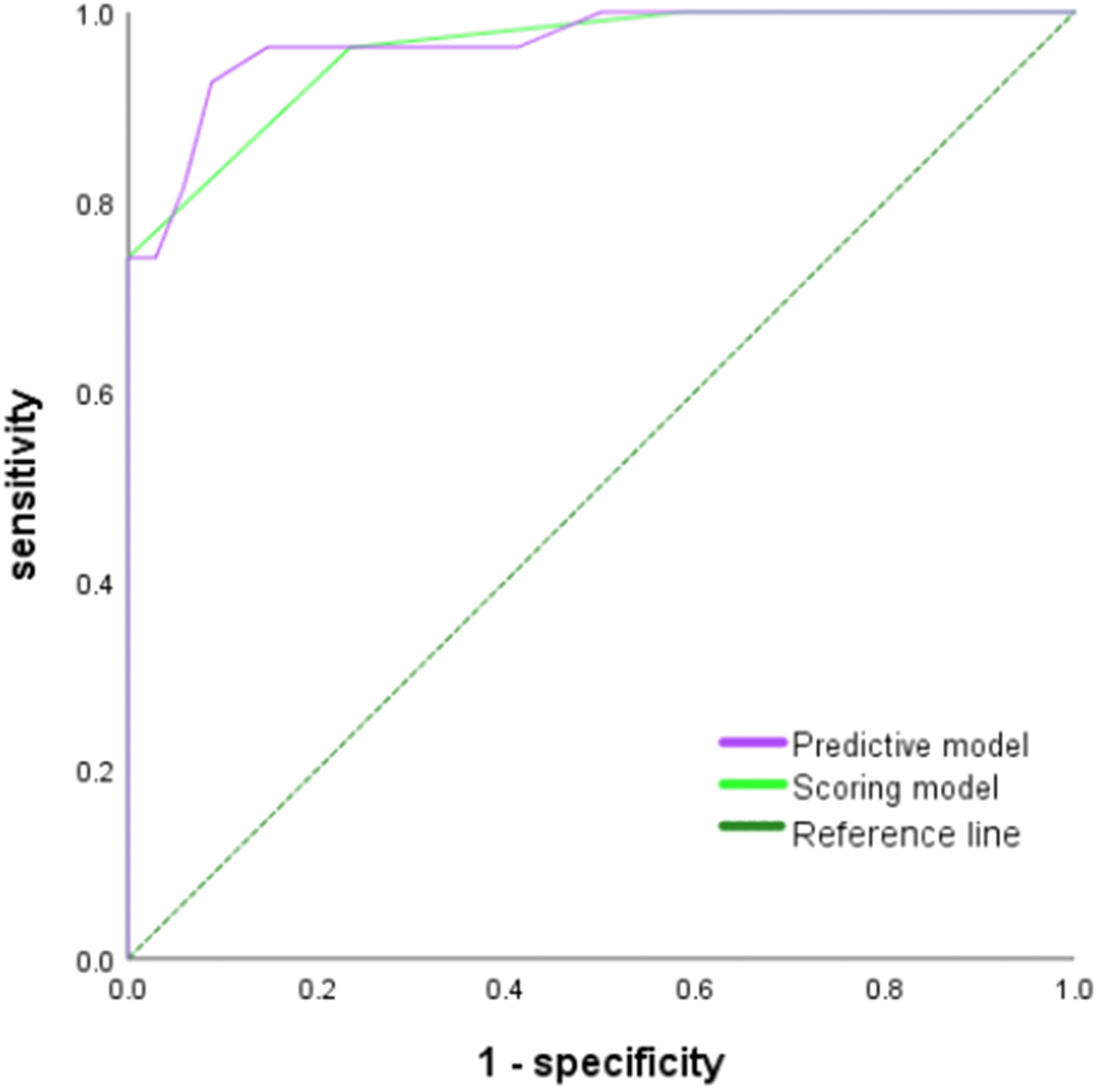
ROC curve of the prediction model and the score model for the diagnosis of ENKTCL in the nose-Waldeyer’s ring of the validation cohort.

In the validation cohort, Hosmer-Lemeshow test of the models presented good calibration (P>0.05). The AUCs of the predictive model and scoring model were 0.967 (95% CI, 0.927-1.000) and 0.959 (95% CI, 0.915-1.000), respectively. The comparison of ROCs between two models in the validation cohort showed no statistical difference (P=0.694> 0.05) testified by DeLong test **(** **Figure 6** **)**. The median score was 8 with extremes of 0 and 16. The specificity was 87.7% and the sensitivity was 86.4% when the cutoff value was 6 points **(** **Table 7** **)**.

## 4 Discussion

NHL is a relatively common extranodal lymphoma in the head and neck, commonly occurred in the nose and Waldeyer’s ring (2). ENKTCL and DLBCL are two major pathological types of NHL, and differences exists between them in terms of histology, clinical features and prognosis (15). In this study, univariate analysis and multivariate logistic regression analysis were conducted to analyze the CT and MRI imaging features, EB virus nucleic acid, and clinical features of 36 patients with ENKTCL and 53 patients with DLBCL. It was revealed that site, blurred edge, high-intense signal on T2WI, gyrus like changes, and positive EB virus nucleic acid were independent predictors for the diagnosis of ENKTCL. Based on the five identified independent predictors, a novel score model for the diagnosis of ENKTCL was established, which is simple, practical. According to the optimal value of AUC, when the value was assigned to 10, the diagnostic accuracy reached 91.7%, which greatly improved the clinically diagnostic accuracy of ENKTCL and the effect of its differentiation with DLBCL. The score model is a new points-scoring system for disease diagnosis pioneered by our research team, and it has been reported in many of our SCI papers and recognized internationally (16–18).

ENKTCL is an invasive subtype of NHL prevalent in East Asia and Latin America, especially in China, Japan and Korea (19). The average age for ENKTCL onset is 44-60 years, and the male to female ratio is about 2:1~3:1 (20). Although extranodal sites such as the nose/paranasal sinuses, skin, gastrointestinal tract, eyes, lungs, and the soft tissues were frequently involved in ENKTCL, ENKTCL mostly occurs in the nose/paranasal sinuses. The correlation between ENKTCL and EB virus infection has been reported (21). Most patients had localized diseases, causing symptoms of nasal congestion, epistaxis and/or destructive mass (2, 21). Univariate analysis in this study showed that the average age of patients with ENKTCL was 55.97 ± 10.13 years, and ENKTCL was more commonly occurred in men, with the main clinical symptoms as nasal obstruction (75.00%), and pharyngeal and facial discomfort (25.00%) (20, 22), while the main clinical symptoms of DLBCL were pharyngeal discomfort (43.40%), nasal obstruction (18.87%), facial discomfort (16.98%), neck discomfort (11.32%), and eye discomfort (9.43%), which were consistent with previous reports (22–26). These clinical symptoms may be related to their sites. ENKTCL, accounting for less than 5% of all extranodal lymphoma in the head and neck, is more likely to occur in the nasal cavity, paranasal sinuses and nasopharynx, while DLBCL, in the proportion of approximately 50% all extranodal lymphoma in the head and neck, is prone to occur in the Waldeyer’s ring. In this study, 72.22% of patients with ENKTCL were tested positive for EB virus nucleic acid, while that of patients with DLBCL only accounted for 24.53%, which was consistent with the proposition that ENKTCL is in a close connection with EB virus (26–28).

Through univariate analysis of the CT and MR imaging features of patients with ENKTCL and DLBCL, statistically significant differences were revealed in site, edge, sinus complex involvement, bone destruction, tonsil enlargement, cervical lymph node enlargement, non-enhancement CT, enhancement CT, signals on T1WI and T2WI, and gyrus like changes. Among the 36 patients with ENKTCL in the nose-Waldeyer’s ring in the present study, the lesions of 26 patients were found in the nasal cavity and paranasal sinuses, with irregular or cast growth, and is easy to invade the nasal septum, turbinate, sinus complex, and surrounding bone. Among the 53 patients with DLBCL, the lesions of 35 patients were found in the Waldeyer’s ring, showing diffuse soft tissue thickening or prominent soft tissue mass, with little infiltration and growth deep into the tissues of pharyngeal wall (4, 23, 29). ENKTCL is commonly presented with diffuse infiltration, while DLBCL is mostly diagnosed with localized mass; the edge of ENKTCL was relatively blurred when in comparison with DLBCL (30). In this study, the occurrence of DLBCL was accompanied more easily by enlargement of cervical lymph nodes and tonsil enlargement (30). In addition, the non-enhancement CT of patients with ENKTCL showed slightly lower density, slightly low-intense signal on T1WI, and high-intense signal on T2WI. After enhanced CT scans, mild to moderate enhancement, and gyrus like changes was observed, which were significant in the sagittal view. The non-enhancement CT of patients with DLBCL showed iso-density, iso-intense signal on T1WI, slightly high-intense signal on T2WI, and mild to moderate enhancement after enhanced CT scans (30, 31). These findings are consistent with those of previous reports.

Previous studies conducted on the diagnosis and differential diagnosis of ENKTCL and DLBCL were mostly descriptive analysis, and the results could not be obtained directly and conveniently. Different from previous studies, the current study established a simpler and more reliable score model for the diagnosis of ENKTCL by assigning values to the imaging features of ENKTCL with significant diagnostic values, following the principle that the higher the diagnostic value, the higher the score. Statistically significant variables identified from univariate analysis were included in multivariate logistic regression analysis, and it was found that site (nose), blurred edge, high-intense signal on T2WI, gyrus like changes, and positive EB virus nucleic acid were independent predictors for the diagnosis of ENKTCL, among which gyrus like changes has not yet been fully studied, while the rest were consistent with the results of several previous studies on the diagnosis of ENKTCL and DLBCL (31). Two studies have reported 5 and 6 imaging features, respectively, helpful in the differential diagnosis of ENKTCL and DLBCL. The proposed imaging features included site, edge, lesion range, bone destruction, cervical lymph node enlargement, positive EB virus nucleic acid, and high-intense signal on T2WI. However, it should be noted that all variables could only be considered as ENKTCL-related factors, not independent predictors for ENKTCL, because they were only analyzed through univariate analysis.

Based on the weighted scores of the regression coefficients of the 5 independent predictors of ENKTCL: 2 points for site, 3 points each for blurred edge and gyrus like changes, and 4 points each for high-intense signal on T2WI and positive EB virus nucleic acid, this study constructed a reliable and easy-to-use points-scoring system that can successfully differentiate ENKTCL from DLBCL. The 0-16 points were further divided into 4 scoring intervals according to the score distribution, and the corresponding diagnostic accuracy of each interval were calculated. When the threshold was ≤ 6 points, all the 34 cases were non-ENKTCL; 7-9 points, about 33.33% were ENKTCL; 10-11 points, about 82.35% were ENKTCL; 12-16 points, 100% were ENKTCL. When the weighted score was ≤ 6 points, and less than two out of these five features could be observed, the score of the patient must be less than 6 points, indicating a greater chance of the patient being diagnosed as DLBCL rather than ENKTCL. In contrast, if the patient scored ≥ 12 points, at least four features could be identified. In this case, the patient should be assuredly diagnosed as ENKTCL rather than DLBCL.

In addition, our study showed that the intra- and interobserver agreements of imaging features (Site、Edge、T2WI、Eyrus like changes) assessments were excellent with all ICC values greater than 0.80 (p-values < 0.001 for all) (**Supplement 1**), suggesting that the repeatability of imaging features assessments were reliable, and the imaging features assessments error would not be a limiting factor for this study.

This study has the following limitations: (1) This is just a retrospective study without groups set for validation, which may lead to bias of the diagnostic efficacy of the score model. (2) The sample size of this study is relatively small, especially for ENKTCL, and inter-group comparison may affect the reliability of the model. Besides, not every score was observed in patients, which may lead to bias in the diagnostic accuracy of ENKTCL. (3) Selection bias may be caused when excluding patients who lacked enhanced CT scans, enhanced MR, or EB virus nucleic acid tests.

In conclusion, we established a novel, simple, practical score model for the diagnosis of ENKTCL by differentiating the imaging features, EB virus nucleic acid, and clinical features of patients with ENKTCL and DLBCL. Five independent predictors were identified: site (nose), blurred edge, high-intense signal on T2WI, gyrus like changes, and positive EB virus nucleic acid. The regression coefficients of the above five independent predictors were weighted, and the weighted scores were 2, 3, 4, 3 and 4, respectively. The overall score of the model was 0-16 points. The score distribution was 0-6 points, 7-9 points, 10-11 points, and 12-16 points, and the corresponding diagnostic accuracy increased gradually, which were 0%, 33.33%, 82.35% and 100%, respectively.

## Data availability statement

The original contributions presented in the study are included in the article/**Supplementary Material**. Further inquiries can be directed to the corresponding authors.

## Ethics statement

The studies involving human participants were reviewed and approved by Ethics Review Committee of The Second Affiliated Hospital of Zhejiang Chinese Medical University. The patients/participants provided their written informed consent to participate in this study.

## Author contributions

J-YX and J-XX contributed to data analysis and manuscript editing. X-SH and YC contributed to the supervision of the whole process. NF and X-ZZ carried out to collect the data of patients. L-MX and Q-PR helped in images analysis. L-YM were responsible for data analysis. All authors contributed to the article and approved the submitted version.
