# Supplementary material for: A diagnostic scoring model of ENKTCL in the nose-Waldeyer’s ring based on logistic regression: Differential diagnosis from DLBCL
Source: Front Oncol. 2023 Feb 15;13:1065440. doi: 10.3389/fonc.2023.1065440 (PMC9975757; doi:10.3389/fonc.2023.1065440)
Supplement: Supplementary file 1 [file Table_1.docx]

**SUPPLEMENT 1 |** The evaluations of the intra- and interobserver agreements in the imaging features in training cohort.

| Parameters | Intraobserver ICC value | Interobserver ICC value |
| --- | --- | --- |
| Site | 0.979（95%CI，0.969-0.986） | 0.965（95%CI，0.947-0.977） |
| Distribution | 0.885（95%CI，0.830-0.923） | 0.835（95%CI，0.759-0.889） |
| Range | 0.933（95%CI，0.900-0.956） | 0.911（95%CI，0.867-0.941） |
| Edge | 0.945（95%CI，0.917-0.964） | 0.893（95%CI，0.840-0.929） |
| Density/Signal | 0.923（95%CI，0.886-0.949） | 0.875（95%CI，0.815-0.916） |
| Hemorrhage | 0.936（95%CI，0.904-0.957） | 0.878（95%CI，0.820-0.918） |
| Cystic | 0.970（95%CI，0.954-0.980） | 0.940（95%CI，0.911-0.960） |
| Pharyngeal recess involvement | 0.902（95%CI，0.855-0.935） | 0.877（95%CI，0.818-0.917） |
| Carotid artery involvement | 0.852（95%CI，0.783-0.900） | 0.809（95%CI，0.724-0.870） |
| Sinus complex involvement | 0.892（95%CI，0.841-0.928） | 0.867（95%CI，0.804-0.911） |
| Bone invasion | 0.910（95%CI，0.866-0.940） | 0.864（95%CI，0.800-0.908） |
| Tonsil enlargement | 0.976（95%CI，0.963-0.984） | 0.927（95%CI，0.891-0.951） |
| Cervical lymph node involvement | 0.977（95%CI，0.965-0.985） | 0.953（95%CI，0.930-0.969） |
| Maximum diameter | 0.992（95%CI，0.986-0.995） | 0.991（95%CI，0.980-0.995） |
| Non-enhancement CT | 0.987（95%CI，0.968-0.993） | 0.984（95%CI，0.968-0.991） |
| Enhancement CT | 0.991（95%CI，0.985-0.994） | 0.989（95%CI，0.982-0.993） |
| D-value | 0.948（95%CI，0.921-0.965） | 0.941（95%CI，0.911-0.961） |
| T1WI | 0.978 （95%CI，0.966-0.985） | 0.933（95%CI，0.900-0.956） |
| T2WI | 0.933（95%CI，0.899-0.955） | 0.888（95%CI，0.835-0.925） |
| Gyrus like changes | 0.867（95%CI，0.805-0.911） | 0.842（95%CI，0.769-0.894） |
| Degree of enhancement | 0.909（95%CI，0.864-0.939） | 0.897（95%CI，0.848-0.931） |
